# Dynamically analyzing cell interactions in biological environments using multiagent social learning framework

**DOI:** 10.1186/s13326-017-0142-0

**Published:** 2017-09-20

**Authors:** Chengwei Zhang, Xiaohong Li, Shuxin Li, Zhiyong Feng

**Affiliations:** 1 School of Computer Science and Technology, Tianjin University, Peiyang Park Campus: No.135 Yaguan Road, Haihe Education Park, Tianjin, 300350 China; 2 School of Computer Computer Software, Tianjin University, Peiyang Park Campus: No.135 Yaguan Road, Haihe Education Park, Tianjin, 300350 China

**Keywords:** Multiagent learning, Cell interaction, Nonlinear dynamic

## Abstract

**Background:**

Biological environment is uncertain and its dynamic is similar to the multiagent environment, thus the research results of the multiagent system area can provide valuable insights to the understanding of biology and are of great significance for the study of biology. Learning in a multiagent environment is highly dynamic since the environment is not stationary anymore and each agent’s behavior changes adaptively in response to other coexisting learners, and vice versa. The dynamics becomes more unpredictable when we move from fixed-agent interaction environments to multiagent social learning framework. Analytical understanding of the underlying dynamics is important and challenging.

**Results:**

In this work, we present a social learning framework with homogeneous learners (e.g., Policy Hill Climbing (*PHC*) learners), and model the behavior of players in the social learning framework as a hybrid dynamical system. By analyzing the dynamical system, we obtain some conditions about convergence or non-convergence. We experimentally verify the predictive power of our model using a number of representative games. Experimental results confirm the theoretical analysis.

**Conclusion:**

Under multiagent social learning framework, we modeled the behavior of agent in biologic environment, and theoretically analyzed the dynamics of the model. We present some sufficient conditions about convergence or non-convergence and prove them theoretically. It can be used to predict the convergence of the system.

## Background

All living systems live in environments that are uncertain and dynamically-changing. However, it is remarkable that these systems survive and achieve their goals by exhibiting intelligent features such as adaption and robustness. Biological system behaviors [1] and human diseases [2] are often the outcome of complex interactions among a very large number of cells and their environments [3, 4].

Similarly, in the multiagent system [5–9], an important ability of an agent is to adjust its behavior adaptively to facilitate efficient coordination among agents in unknown and dynamic environments. If we regard the cells in the biological system as the agents in the multiagent system, we can analyse the cells’ behavior using the theory of multiagent system. So understanding collective decision made by such intelligent multiagent system is an interesting research topic not only for artificial intelligent but also for biology. The conclusion of the theoretical analysis can be applied to the research of biology, for example, the results of convergence can be used for explaining the phenomenon of cell’s group behaviour.

Now, computational methods have been widely used to solve biological problems [10, 11]. Many researchers have investigated biological systems which are composed of cells and their environments via modeling and simulation [1, 12]. There are two principal approaches: population based modeling and discrete agent based modeling. Population based modeling approximates the cells within any grid box by a set of variables associated with the grid box [13, 14]. Discrete agent based modeling maps each cell to a discrete simulation entity [13, 15, 16].

We use multiagent learning techniques to model the behaviors of each cell agent, which is an important technique to achieve efficient coordination in multiagent system area [9, 17–19]. Until now, significant amount of efforts have been devoted to develop effective learning techniques for different multiagent interaction environments [20–23]. In the multiagent environments, each agent interacts with the agent selected from its neighborhood randomly each round, and updates its strategy based on the feedback in the current round. To describe the behavior of an agent, one common line of researches is to extend existing reinforcement learning techniques in single-agent environment to multiple-agent interaction environment. However, due to the violation of Markov property, the existing theoretical guarantees do not hold any more in multiagent environment. It is important and challenging for us to model the multi-agent environment and analyse the learning dynamics of multiagent environments.

This paper presents a social learning framework to simulate the dynamics of multiagent system in biological environment and a theoretical analysis of the learning dynamics of this model is also given. The analysis results shed lights on how and when the consistent knowledge in terms of equilibrium can be or not be evolved among the population of agents. In the social learning framework, all agents play *PHC* strategy [24] for decision making, and use a weighted graph model for neighbor selection. In the part of theoretical analysis, we present a theoretical model to analyze the learning dynamics of the learning framework. The purpose of analysing the learning dynamics is to judge whether the learning algorithm that the agent adopt can converge or not. The intention behind is that convergence to an equilibrium has been the most commonly accepted goal to pursue in multiagent learning literature. Firstly, we model the overall dynamics among agents as a system of differential equations. Then, some conditions are proved to be the sufficient condition of convergence or non-convergence. It can be used to predict the convergence of the system. Finally, we estimate the prediction through simulation experiment. The experimental results confirm the predictive outcomes of our theoretical analysis.

The remainder of the paper is organized as follows. “Method” section first reviews normal-form game and the basic gradient ascent approach with a GA-based algorithm named *PHC*, and then introduces the multiagent learning framework where all the agents are *PHC* learners. In the “Result and discussion” section, we present the theoretical model of the learning dynamics of agents, and prove convergence and non-convergence conditions by analyze geometrical behaviors of the hybrid dynamic system in the help of nonlinear dynamic theory. In the “Experimental simulation” section, we evaluate the predictive ability of our theoretical model by comparing it with the simulation results. Lastly we conclude the paper and point out future directions in “Conclusion” section.

## Method

### Notation and definition

#### Normal-form games

In a two-player, two-action, general-sum normal-form game, the payoff for each player *i*∈{*k,l*} can be specified by a matrix as follows,

1 $$ R_{i}=\left[ \begin{array}{ll} r_{i}^{11} &r_{i}^{12} \\ r_{i}^{21}&r_{i}^{22} \end{array} \right]  $$

Each player *i* selects an action simultaneously from its action set *A*_*i*_={1,2}, and the payoff of each player is determined by their joint actions. For example, if player *k* selects the pure strategy of action 1 while player *l* selects the pure strategy of action 2, then player *k* receives a payoff of $r_{k}^{12}$ and player *l* receives the payoff of $r_{l}^{21}$.

Apart from pure strategy, each player can also employ a mixed strategy to make decisions. A mixed strategy can be represented as a probability distribution over the action set and a pure strategy is a special case of mixed strategies. Let *p*_*k*_∈[0,1] and *p*_*l*_∈[0,1] denote the probability of choosing action 1 by player *k* and player *l*, respectively. Given a joint mixed strategy profile (*p*_*k*_,*p*_*l*_), the expected payoffs of player *l* and player *k* can be computed as follows,

2 $$\begin{array}{*{20}l} V_{k}\left(p_{k},p_{l} \right)=&r_{k}^{11}p_{k}p_{l}+r_{k}^{12}p_{k}\left (1-p_{l}\right)+r_{k}^{21}\left(1-p_{k}\right)p_{l}\\ &+r_{k}^{22}\left (1-p_{k}\right)\left(1-p_{l}\right) \end{array} $$

3 $$\begin{array}{*{20}l} V_{l}\left (p_{k},p_{l} \right)=&r_{l}^{11}p_{k}p_{l}+r_{l}^{21}p_{k}\left (1-p_{l}\right)+r_{l}^{12}\left(1-p_{k}\right)p_{l}\\ &+r_{l}^{22}\left (1-p_{k}\right)\left(1-p_{l}\right) \end{array} $$

A strategy profile is a Nash Equilibrium (NE) if no player can get a better expected payoff by changing its current strategy unilaterally. Formally, $\left (p_{k}^{*},p_{l}^{*} \right)\in \left [0,1 \right ]^{2}$ is a NE, iff $V_{k}\left (p_{k}^{*},p_{l}^{*} \right)\geq V_{k}\left (p_{k},p_{l}^{*} \right)$ and $V_{l}\left (p_{k}^{*},p_{l}^{*} \right)\geq V_{l}\left (p_{k}^{*},p_{l}\right)$ for any (*p*_*k*_,*p*_*l*_)∈[0,1]^2^.

#### Gradient ascent (GA) and *PHC* algorithm

When a game is repeatedly played, an individually rational agent updates its strategy with the propose of maximizing its expected payoff. We know that the gradient direction is the fastest increasing direction, thus it is a well-deserved way to model the behavior of agent using gradient ascent algorithm. Agent *i* that employs GA-based algorithm updates its policy towards the direction of its expected reward gradient, which is shown in the following equations.

4 $$\begin{array}{@{}rcl@{}} \Delta p_{i}^{\left(t+1\right)}\leftarrow \eta \frac{\partial V_{i}\left (p^{(t)}\right)}{\partial p_{i}} \end{array} $$

5 $$\begin{array}{@{}rcl@{}} p_{i}^{\left(t+1\right)}\leftarrow \Pi_{\left [0,1\right ]}\left (p_{i}^{(t)}+\Delta p_{i}^{\left(t+1\right)}\right) \end{array} $$

The parameter *η* is the size of gradient step. *Π*_[0,1]_ is the projection function mapping the input value to the valid probability range of [0,1], which is used for preventing the gradient from moving the strategy out of the valid probability space. Formally, we have

6 $$ \Pi_{\left [0,1 \right]}(x)={argmin}_{z\in\left[0,1\right]}\left|x-z\right|.  $$

To simplify the notation, let us define $u_{i}=r_{i}^{11}+r_{i}^{22}-r_{i}^{12}-r_{i}^{21}$ and $c_{i}=r_{i}^{12}-r_{i}^{22}$. For the two-player case, the Eqs. 4 and 5 can be represented as follows,

7 $$ p_{k}^{\left(t+1\right)}\leftarrow \Pi_{\left [0,1\right]}\left(p_{k}^{(t)}+\eta\left(u_{k}p_{l}^{(t)}+c_{k}\right)\right)  $$

8 $$ p_{l}^{\left(t+1\right)}\leftarrow \Pi_{\left [0,1\right]}\left(p_{l}^{(t)}+\eta\left(u_{l}p_{k}^{(t)}+c_{l}\right)\right).  $$

In the case of infinitesimal size of gradient step (*η*→0), the learning dynamics of the agent can be modeled as a system of differential equations. Further, it can be analyzed using dynamic system theory [25]. It is proved that the strategies of all agents will converge to a Nash equilibrium, or if the strategies do not converge, agents’ average payoff will converge to the average payoff of Nash equilibrium [26]. The policy hill-climbing algorithm (*PHC*) is a combination of gradient ascent algorithm and Q-learning where each agent *i* adjusts its policy *p* to follow the gradient of expected payoff (or the value function *Q*). It is shown in the Algorithm 1.



Here, *α*∈(0,1] and *δ*∈(0,1] are learning rate, and *Q* values are maintained just as in normal *Q*-learning. The policy is improved by increasing the probability of selecting the highest valued action based on the learning rate *δ*.

### Modeling multiagent learning

Under the multiagent social learning framework with *N* agents, each agent interacts with one of its neighbors selected randomly from its neighborhood each round. The neighborhood of each agent is determined by its underlying network topology. The interaction between each pair of agents is modeled as a two-player normal-form game. During each interaction, each agent selects its action following a specified learning strategy, which is updated repeatedly based on the feedback from the environment at the end of interaction. The framework is presented in Algorithm 2.



We use graph *G*=(*V,E*) to model the underlying neighborhood network, which is composed by *N*=|*V*| agents. The edges *E*={*e*_*ij*_}, *i,j*∈*V* represent social contacts among agents, where *e*_*ij*_ denotes the probability that agent *i* chooses agent *j* to interact with. We have ${\sum \nolimits }_{j\in V}{{{e}_{ij}}=1}\wedge {{e}_{ii}}=0$. Here, we propose an adaptive strategy for agents to make their decisions in social learning framework with PHC learning strategy, which is shown in Algorithm 3.



## Result and discussion

### Analysis of the multiagent Learning Dynamics

In this section, we present a theoretical model to estimate and analyze the learning dynamics of the above multiagent learning framework in Algorithm 3. We extend notations in section to the multiagent environment. Without loss of generality, we consider the case with two-action only.

Assume that the payoff that an agent receives only depends on the joint action, then the payoff for agent *i*∈*V* can be defined as a fixed matrix *R*_*i*_,

9 $$  R_{i}=\left[ \begin{array}{ll} r_{i}^{11} &r_{i}^{12} \\ r_{i}^{21}&r_{i}^{22} \end{array}\right]  $$

where $r_{i}^{mn}$ denotes the payoff received by agent *i* when *i* selects action *m* and its neighbor selects *n*. Here, we use the *p*_*i*_ to denote the probability that the player *i* selects action 1. Then the mixed strategy (*p*_1_,*p*_2_,…,*p*_*N*_) in multiagent framework can be considered as a point in ${\mathbb {R}}^{N}$ constrained to the unit square. The expected payoff *V*_*i*_(*p*_1_,*p*_2_,…,*p*_*N*_) of player *i* can be computed as follows,

10 $$  \begin{aligned} &{V}_{i}({{p}_{1}},{{p}_{2}},\ldots,{{p}_{n}}) \\ =&{\sum\nolimits}_{j\in V}{e_{ij}V_{i,j}\left(p_{i},p_{j}\right)}\\ =&{{u}_{i}}{{p}_{i}}{\sum\nolimits}_{j\in V}{{{e}_{ij}}{{p}_{j}}}+{{c}_{i}}{{p}_{i}}+\left(r_{i}^{21}-r_{i}^{22}\right){{p}_{j}}+r_{i}^{22} \\ \end{aligned}  $$

where $u_{i}=r_{i}^{11}+r_{i}^{22}-r_{i}^{12}-r_{i}^{21}$, $c_{i}=r_{i}^{12}-r_{i}^{22}$, $V_{i,j}\left (p_{i},p_{j}\right)=r_{i}^{11}p_{i}p_{j}+r_{i}^{12}p_{i}\left (1-p_{j}\right)+r_{i}^{21}\left (1-p_{i}\right)p_{j}+r_{i}^{22}\left (1-p_{i}\right)\left (1-p_{j}\right)$, and *e*_*ij*_ is the probability that the agent *i* selects agent *j* to interact with.

Each agent *i* updates its strategy in order to maximize the value of *V*_*i*_. Recall the Eqs. 4 and 5, we can obtain

11 $$ \begin{aligned} p_{i}^{(k+1)}&=\prod\nolimits_{\Delta }{\left[ p_{i}^{(k)}+\eta {{\partial }_{{{p}_{i}}}}{{V}_{i}}({{p}_{1}},{{p}_{2}},\ldots,{{p}_{N}}) \right]} \\ &=\prod\nolimits_{\Delta }{\left[ p_{i}^{(k)}+\eta \left({{u}_{i}}{\sum\nolimits}_{j\in V}{{{e}_{ij}}{{p}_{j}}}+{{c}_{i}} \right) \right]} \end{aligned}   $$

where parameter *η* is the size of gradient step.

As *η*_*p*_→0, it is straightforward that the Eq. 11 becomes differential equation. Considering the step size to be infinitesimal, the unconstrained dynamics of the all players’ strategies can be modeled by the following differential equations.

12 $$  {{{\dot p}_{i}} = {u_{i}}{\sum\nolimits}_{j \in V} {{e_{ij}}{p_{j}}} + {c_{i}},}\quad{i \in \{ 1,2,\ldots,N\} }  $$

Equation 12 can be simplified as follows using some notation,

13 $$  \dot{P}=UEP+C  $$

where *P*=(*p*_1_,*p*_2_,…,*p*_*N*_)^*T*^, $\dot {P}={{({{\dot {p}}_{1}},{{\dot {p}}_{2}},\ldots,{{\dot {p}}_{N}})}^{T}}$ and *C*=(*c*_1_,*c*_2_,…,*c*_*N*_)^*T*^. The matrix *U*=*diag*(*u*_1_,*u*_2_,…,*u*_*N*_) is the diagonal matrix generated by (*u*_1_,*u*_2_,…,*u*_*N*_).

For the constrained dynamics of the strategies, we can model it as the following equations,

14 $$  \left\{ \begin{array}{ll} {{{\dot{p}}}_{i}}=0 & {{p}_{i}}=0\wedge {{G}_{i}}\le 0 \\ {{{\dot{p}}}_{i}}=0 & {{p}_{i}}=1\wedge {{G}_{i}}\ge 0 \\ {{{\dot{p}}}_{i}}={{G}_{i}} & otherwise \\ \end{array} \right.  $$

where ${G}_{i}={{u}_{i}}{\sum \nolimits }_{j\in V}{{{e}_{ij}}{{p}_{j}}}+{{c}_{i}}$.

Notice that Eq. 14 is a hybrid system composed of two parts: a series of continuous linear differential dynamic systems in the respective domain space and a switch mechanism between differential dynamic systems when dynamic touch the boundary. Generally, it is hard to obtain a complete conclusion by analyzing dynamics of a general hybrid system, even though the differential system is linear. But we can still find some convergence and non-convergence conditions under certain instances(i.e., Eq. 14).

### Non-convergence condition of the multiagent learning framework

According to the above definition, we have the following general result under which non-convergence is guaranteed.

#### **Theorem 1**

In an *N* agent, two-action, integrated general sum game, every player follows the constrained dynamics of the strategy we defined in Eq. 14. If the following two conditions are met,

1. There exists a point $P^{*}=\left (p_{1}^{*},p_{2}^{*},\ldots,p_{N}^{*}\right)\in \left (0,1\right)^{N}$, that *U* *E* *P* ^∗^+*C*=0,
2. There exists a pair of pure imaginary eigenvalues of matrix *UE*,

then there exists a set $\mathbb {P}\subset {{[0,1]}^{N}}$, that the solution of the initial value problem of Eq. 14 with $P(0)\in \mathbb {P}$ can not converge.

#### *Proof*

Considering the complexity of the hybrid system represented by Eq. 14, we begin with the unconstrained ones. Based on the theorems of differential equations dynamical systems [25], we calculate the analytic solution of Eq. 13. Homogenizing the in-homogeneous equation by substituting *P* with *P*=*X*+*P* ^∗^, where *U* *E* *P* ^∗^+*C*=0, we get

$$\dot{X}=UEX. $$

Here, *UE* is an *N*×*N* matrix, then there is a invertible matrix *T*=(*v*_1_,…,*v*_*N*_) that can transform *UE* into *J*,

$${{T}^{-1}}UET=J=\left[ \begin{array}{lll} {{J}_{1}} & \cdots & {} \\ \vdots & \ddots & \vdots \\ {} & \cdots & {{J}_{m}} \\ \end{array} \right] $$

The *J*_*i*_ is a square matrix and its form is one of the following two,

$$(1)\left[ \begin{array}{cccc} \lambda & 1 & \cdots & {} \\ {} & \lambda & 1 & {} \\ \vdots & {} & \ddots & \vdots \\ {} & {} & \cdots & \lambda \\ \end{array} \right] (2)\left[ \begin{array}{cccc} {{D}_{2}} & {{I}_{2}} & \cdots & {} \\ {} & {{D}_{2}} & {{I}_{2}} & {} \\ \vdots & {} & \ddots & \vdots \\ {} & {} & \cdots & {{D}_{2}} \\ \end{array} \right] $$

where ${{D}_{2}}=\left [ \begin {array}{ccc} \alpha & \beta \\ -\beta & \alpha \\ \end {array} \right ]$, ${{I}_{2}}=\left [ \begin {array}{ccc} 1 & 0 \\ 0 & 1 \\ \end {array} \right ]$, $\alpha,\beta,\lambda \in \mathbb {R}$ and *β*≠0. Here, *J* is the Jordan normal form of matrix *UE*. *J*_*i*_ is the Jordan block corresponding to *λ*_*i*_, which is a repeated eigenvalue of *UE* with multiplicity *n*_*i*_. If eigenvalue *λ*_*i*_ is a real number, then *J*_*i*_ is in the form (1), else *J*_*i*_ is in the form (2). Suppose that *λ*_1_,…,*λ*_*k*_ are matrix *UE*’s real eigenvalues, and *λ*_*k*+1_,…,*λ*_*m*_ is matrix *UE*’s complex eigenvalues, then we have *n*_1_+…+*n*_*k*_+2(*n*_*k*+1_+…*n*_*m*_)=*N*.

Then the analytic solution of function $\dot {X}=UEX$ with initial value *X*(0) will be

$$ X(t)=\exp\left(tUE \right)X(0)=T\left[ \begin{array}{ccc} {{e}^{t{{J}_{1}}}} & {} & {} \\ {} & \ddots & {} \\ {} & {} & {{e}^{t{{J}_{m}}}} \\ \end{array} \right]{{T}^{-1}}X(0).   $$

Using the notation *Y*(*t*)=*T* ^−1^ *X*(*t*), we have

$$ Y(t)=\exp \left(tJ \right)Y(0)=\left[ \begin{array}{ccc} {{e}^{t{{J}_{1}}}} & {} & {} \\ {} & \ddots & {} \\ {} & {} & {{e}^{t{{J}_{m}}}} \\ \end{array} \right]Y(0).   $$

Suppose that *λ*_*k*_=*β* *i* is a pure imaginary eigenvalue of *UE* with multiplicity *n*_*k*_, so ${{\bar {\lambda }}_{k}}=-\beta {i}$ is an eigenvalue of *UE* with multiplicity *n*_*k*_. Then *J* has a block *J*_*k*_, ${{J}_{k}}={{\left [ \begin {array}{cccc} {{D}_{2}} & {{I}_{2}} & \cdots & {} \\ {} & {{D}_{2}} & {{I}_{2}} & {} \\ \vdots & {} & \ddots & \vdots \\ {} & {} & \cdots & {{D}_{2}} \\ \end {array} \right ]}}$, where ${{D}_{2}}=\left [ \begin {array}{cc} 0 & \beta \\ -\beta & 0 \\ \end {array} \right ]$.

Due to ${{e}^{t{{D}_{2}}}}=\exp \left (t\left [ \begin {array}{cc} 0 & \beta \\ -\beta & 0 \\ \end {array} \right ] \right)=\left [ \begin {array}{cc} \cos \beta t & \sin \beta t \\ -\sin \beta t & \cos \beta t \\ \end {array} \right ]$, there must exist a pair of items about vector *Y*(*t*) as follows.

$$\left\{ \begin{array}{ll} {{y}_{i}}(t)={{y}_{i}}(0)\cos \beta t+{{y}_{i+1}}(0)\sin \beta t \\ {{y}_{i+1}}(t)=-{{y}_{i}}(0)\cos \beta t+{{y}_{i+1}}(0)\sin \beta t \\ \end{array} \right. $$

If *y*_*i*_(0)≠0∨*y*_*i*+1_(0)≠0, then Eq. 14 has a periodic solution. Let *v*_*i*_ and *v*_*i*+1_ to denote eigenvector of *T*=(*v*_1_,…,*v*_*N*_) corresponding to *λ*_*k*_ and $\bar {\lambda }_{k}$, respectively. Note that *X*(*t*)=*T* *Y*(*t*), then the solution of Eq. 13 with the initial value *P*(0)∈*S* is cyclical, where

$$S=\left\{ P\in {{[0,1]}^{N}}|P={{k}_{1}}{{v}_{1}}+{{k}_{2}}{{v}_{2}}+{{P}^{*}},{{k}_{1}},{{k}_{2}}\in \mathbb{R} \right\}. $$

Because of *P* ^∗^∈(0,1)^*N*^, there must exists a *ε*>0 for the deleted neighborhood $\mathbb {B}({{P}^{*}};\varepsilon)\subset \left (0,1\right)^{N}$ of *P* ^∗^,

$$\mathbb{B}({{P}^{*}};\varepsilon)=\left\{ x\in {{\mathbb{R}}^{N}}|0<||x-{{P}^{*}}|{{|}_{2}}<\varepsilon \right\}\subset {{(0,1)}^{N}} $$

Let $\mathbb {P}$ denote $S\bigcap \mathbb {B}({{P}^{*}};\varepsilon)$, the solution of the Eq. 14 with any initial value belongs to $\mathbb {P}$ is cyclical, which means the algorithm corresponding to the Eq. 14 can not converge. □

Theorem 1 shows that there exist some situations in which the agents fail to converge under the multiagent social learning framework. Before giving the details of those situations, we need to introduce the following notations first.

According to the theorem 1, *T* is the transformation matrix for *T* ^−1^ *U* *E* *T*=*J*, *T*=(*v*_1_,*v*_2_,…,*v*_*N*_). Let $\phantom {\dot {i}\!}v_{j1},v_{j2},\ldots,v_{{jn}_{j}}$ denote eigenvectors associated to eigenvalue *λ*_*j*_, *j*=1,2,…,*m*. According to properties of the matrix transformations [27], $\phantom {\dot {i}\!}v_{j1},v_{j2},\ldots,v_{{jn}_{j}}$ are linearly independent. Classify column vectors of the transformation matrix *T* into three parts corresponding to *λ*, *V*_1_={*v*_*i*_|*R* *e*(*λ*_*i*_)<0}, *V*_2_={*v*_*i*_|*R* *e*(*λ*_*i*_)=0} and *V*_3_={*v*_*i*_|*R* *e*(*λ*_*i*_)>0}. Now we are ready to give the precise description of the subspace where the agents fail to converge, which is summarized in the following theorem.

#### **Theorem 2**

If Eq. 14 meets both conditions of Theorem 1, and *λ*_*k*_=*β* *i*, $\overline {{\lambda _{\mathrm {k}}}} = - \beta i$ are a pair of pure imaginary eigenvalues of *UE*, then there exists a pair of vectors *v*_*k*_,*v* *k*′∈*V*_2_, *ε*>0, and a set $\mathbb {P}=\mathbb {S}\cap \mathbb {B}({{P}^{*}};\varepsilon)$, where

$${} \mathbb{S}\,=\,\left\{ P \in {[0,1]}^{N}|P \,=\, X + {P^{*}},X \in span({V_{1}} \cup \{ {v_{k}},{v_{k}^{'}}\}) \right\}, $$

$$\mathbb{B}({{P}^{*}};\varepsilon)=\left\{ {x \in {\mathbb{R}^{N}}|0 < ||x - {P^{*}}|{|_{2}} < \varepsilon} \right\} \subset {[0,1]^{N}}, $$

that the solution of the initial value problem of the Eq. 14 with $P(0)\in \mathbb {P}$ can’t convergence.

#### *Proof*

According to Theorem 1, we have the solution of the initial value problem that the Eq. 14 with $ P(0)\in S\cap \mathbb {B}({{P}^{*}};\varepsilon)$ can not convergence. Here

$$ S=\left\{ {P \in {{[0,1]}^{N}}|P = X + {P^{*}},X \in span(\{ {v_{k}},{v_{k}'}\})} \right\} $$

For the eigenvalue *λ*_*i*_ associated to vector *v*_*i*_∈*V*_1_, there are *R* *e*(*λ*_*i*_)<0. According to conclusions of bifurcation theory [25], the subspace *span*(*V*_1_) is a stable submanifold of the unconstrained dynamics (13), which means every trajectory start from *S* ^′^ will eventually convergence to *P* ^∗^, where

$$ S'=\left\{ {P \in {{[0,1]}^{N}}|P = X + {P^{*}},X \in span(V_{1})} \right\}. $$

Then trajectories start from $\mathbb {S}$ will eventually convergence to *S*, thus we got the final conclusion that the solution of the initial value problem of the Eq. 14 with $P(0)\in \mathbb {P}$ can’t convergence. □

Note that Theorem 1 and 2 are just sufficient conditions of non-convergence.

### Convergence condition of the multiagent learning framework

In most cases, the conditions that guarantee the convergence of a algorithm are more valuable.

#### **Theorem 3**

In an *N* agent, two-action, integrated general sum game, every player follows the constrained dynamics of the strategy we defined in Eq. 14. If the following two conditions are met,

1. There exists a point $P^{*}=\left (p_{1}^{*},p_{2}^{*},\ldots,p_{N}^{*}\right)\in \left (0,1\right)^{N}$, that *U* *E* *P* ^∗^+*C*=0,
2. All of the eigenvalues of matrix *UE* has negative real part,

then all the solutions of the initial value problem of Eq. 14 with *P*(0)∈[0,1]^*N*^ will converge eventually.

#### *Proof*

The conclusion is obvious. It is known that the construction of the linear dynamic system is stable. If all eigenvalues of matrix *UE* have negative real part, then point *P* is a stable equilibrium point. It means that all the solutions of the initial value problem of the Eq. 14 with *P*(0)∈[0,1]^*N*^ will converge to *P*. □

Theorem 3 proposes a sufficient condition to identify the convergence of dynamic in Eq. 14. We know that it is hard to calculate eigenvalues of a matrix with high dimensional. Here, we propose a more realistic convergence condition which is suitable for multiagent learning framework shown in Algorithm 3.

#### **Theorem 4**

In an *N* agent, two-action, integrated general sum game, every player follows the constrained dynamics of the strategy we defined in Eq. 14. If matrix *UE* is symmetrical, then all the solution of the initial value problem of Eq. 14 with *P*(0)∈[0,1]^*N*^ will converge eventually.

#### *Proof*

It is known that the eigenvalues of real symmetric matrix are real numbers [27]. We analyze all the cases of Eq. 14 when all of the eigenvalues of matrix *UE* are real:

1. There exists a point $P^{*}=\left (p_{1}^{*},p_{2}^{*},\ldots,p_{N}^{*}\right)\in \left (0,1\right)^{N}$, that *U* *E* *P* ^∗^+*C*=0.
2. There are no such a point, that *U* *E* *P* ^∗^+*C*=0.

For case 1), if all eigenvalues of matrix *UE* are negative number, then point *P* is a stable equilibrium points; otherwise, all the solutions of the initial value problem of the hybrid system with *P*(0)∈[0,1]^*N*^ will move away from *P* toward boundary of the hybrid system [25]. Because the domain of hybrid system represented by 14 has boundary(i.e., *P*(*t*)∈[0,1]^*N*^), then there must exists a point *P* ^′^=(*p*1′,…,*p* *N*′)^*T*^ in the boundary of the domain, where $({{p^{\prime }}_{i}}=0\wedge {{G}_{i}}\le 0)\vee ({{p^{\prime }}_{i}}=1\wedge {{G}_{i}}\ge 0)$ for all *i*∈*V*. The dynamic *P*(*t*) will converge to *P* ^′^ eventually.

Similarly, we can find a point $P^{\prime }=\left (p^{\prime }_{1},\ldots,p^{\prime }_{N}\right)^{T}$ in the boundary of the hybrid system domain in case 2) and the dynamic *P*(*t*) will converge to *P* ^′^ eventually. The theorem must hold. □

Based on conclusions of Subsections Non-convergence condition of the multiagent learning framework and Convergence condition of the multiagent learning framework, we can determine the learning dynamics of any cases we defined in Eqs. 14 and 13. However, the computational complexity may be prohibitive when the model size becomes too large. In the next section, we consider a special case under an interesting network structure which can be analyzed with relatively light computational complexity for any network size.

### The simplest case whose underlying topology is a ring

We consider the case when the underlying topology is a ring, and each agent only interacts with the neighbor on its right-hand side in each interaction. As defined in the previous section, the adjacency matrix *E* is

$$E = {\{ {e_{ij}}\}_{N \times N}},i,j \in \{ 1,2,\ldots,N\}, $$

where ${e_{ij}} = \left \{ {\begin {array}{*{20}{c}} 1& j = (i + 1)_{} {mod}_{} N\\ 0&{else} \end {array}}. \right.$

According to Eq. 14, the constrained dynamics of this special case can be modeled as follows:

15 $$  \left\{ \begin{array}{ll} {{{\dot{p}}}_{i}}=0 & {{p}_{i}}=0\wedge {{G}_{i}}\le 0 \\ {{{\dot{p}}}_{i}}=0 & {{p}_{i}}=1\wedge {{G}_{i}}\ge 0 \\ {{{\dot{p}}}_{i}}={{G}_{i}} & otherwise \\ \end{array} \right.  $$

where *G*_*i*_=*u*_*i*_ *p*_*i*+1_+*c*_*i*_, *i*={1,2,…,*N*−1}, and *G*_*N*_=*u*_*N*_ *p*_1_+*c*_*N*_. Through analyzing the dynamics of this model, we have the following conclusion.

#### **Theorem 5**

In an N-player, two-action, integrated general-sum game, every agent follows the constrained dynamics of the model in Eq. 15. If one of the agents converges to a strategy, then every agent will converges eventually.

#### *Proof*

Suppose agent *k* converges at some time, according to the definition, its strategy *p*_*k*_ will be a constant. In Eq. 15, we have *G*_*k*−1_=*u*_*k*−1_ *p*_*k*_+*c*_*k*−1_ be a constant, which means convergence of player *k* implies convergence of player *k*−1. By induction, every agent will converge eventually. □

According to the above theorem, we can easily obtain the following proposition.

#### **Proposition 1**

In Eq. 15, if there exists a dominant strategy for some players, then their strategies will asymptotically converge to a Nash equilibrium.

According to the above conclusion, finally we present the following unconvergence result.

#### **Theorem 6**

In an *N* agent, two-action, integrated general sum game, every player follows the constrained dynamics of the strategy we defined in Eq. 15. If every player has no dominant strategy, and met one of the following conditions,

1. *N*=4*k*, $k\in \mathbb {N}$ and $\prod _{i=1}^{N}u_{i}>0$.
2. *N*=4*k*+2, $k\in \mathbb {N}$ and $\prod _{i=1}^{N}u_{i}<0$.

then there exists a set $\mathbb {P}\subset \left [0,1\right ]^{N}$ that the solution of the initial value problem of the Eq. 15 with $P(0)\in \mathbb {P}$ can’t converge.

#### *Proof*

According to the definitions above, the payoff matrix of player *i* is

$$ R_{i}=\left[ \begin{array}{ll} r_{i}^{11} &r_{i}^{12} \\ r_{i}^{21}&r_{i}^{22} \end{array} \right],i\in \{1,2,\ldots,N\}, $$

and $u_{i}=r_{i}^{11}+r_{i}^{22}-r_{i}^{12}-r_{i}^{21}$, $c_{i}=r_{i}^{12}-r_{i}^{22}$. Then we have

16 $$ \begin{aligned} &u_{i}c_{i} \\ =&\left(r_{i}^{11}+r_{i}^{22}-r_{i}^{12}-r_{i}^{21}\right)\left (r_{i}^{12}-r_{i}^{22} \right)\\ =&\left(r_{i}^{11}-r_{i}^{21}\right)\left(r_{i}^{12}-r_{i}^{22}\right) -\left(r_{i}^{12}-r_{i}^{22}\right)^{2}\\ \end{aligned}  $$

Since every agent has no dominant strategy, we have $\left (r_{i}^{11}-r_{i}^{21}\right)\left (r_{i}^{12}-r_{i}^{22}\right)<0$.

Thus we have *u*_*i*_ *c*_*i*_<0, and

$$\frac{c_{i}}{u_{i}} =-\frac{\left(r_{i}^{12}-r_{i}^{22}\right)}{\left(r_{i}^{11}-r_{i}^{21}\right)-\left(r_{i}^{12}-r_{i}^{22}\right)}=\frac{1}{1+\frac{\left(r_{i}^{21}-r_{i}^{11}\right)}{\left(r_{i}^{12}-r_{i}^{22}\right)}}. $$

Set $p_{i}^{*}=-\frac {c_{i}}{u_{i}}$ and $P^{*}=\left (p_{1}^{*},p_{2}^{*},\ldots,p_{N}^{*}\right)^{T}$, then we have *P* ^∗^∈(0,1)^*N*^ and *U* *E* *P*+*C*=0. Considering the Eq. 15, by calculating the eigenvalue of matrix *UE*, we have

$$\lambda^{N}=u_{1}u_{2}\ldots{u}_{N}=\prod_{i=1}^{N}u_{i}. $$

If *N*=4*k*, $k\in \mathbb {N}$ and $\prod _{i=1}^{N}u_{i}>0$, then matrix *UE* has a pair of pure imaginary eigenvalue. Otherwise, if *N*=4*k*+2, $k\in \mathbb {N}$ and $\prod _{i=1}^{N}u_{i}<0$, then matrix *UE* has a pair of pure imaginary eigenvalue. According to Theorem 1, there exists a set $\mathbb {P}\subset \left [0,1\right ]^{N}$ that the solution of the initial value problem of Eq. 15 with $P(0)\in \mathbb {P}$ can not convergence. □

## Experimental simulation

In this section, we compare the empirical dynamics of the multiagent social learning framework composed by *PHC* learners with theoretical prediction of our hybrid dynamic model. We perform two experiments that satisfy the Theorem 1 and 4, respectively.

### A non-convergence multiagent Game

In this subsection, we consider a 4-player, two-action game. The game is defined as follows,

$${} {{R}_{1}}=\left[ \begin{array}{ll} 1 & 0 \\ 0 & 1 \\ \end{array} \right],{{R}_{2}}=\left[ \begin{array}{ll} 1 & 0 \\ 0 & 1 \\ \end{array} \right],{{R}_{3}}=\left[ \begin{array}{ll} 1 & 0 \\ 0 & 1 \\ \end{array} \right],{{R}_{4}}=\left[ \begin{array}{ll} 1 & 0 \\ 0 & 1 \\ \end{array} \right] $$

$$E=\left[ \begin{array}{cccc} 0 & 1/2 & 0 & 1/2 \\ 1/2 & 0 & 1/2 & 0 \\ 0 & 1/2 & 0 & 1/2 \\ 1/2 & 0 & 1/2 & 0 \\ \end{array} \right] $$

Metrix *R*_*i*_,*i*∈{1,2,3,4} is the payoff matrix of agent *i*, and element *e*_*ij*_ of matrix *E* is the probability that player *i* selects player *j* in each interaction. In this game, we have *u*_1_=*u*_3_=2, *u*_2_=*u*_4_=−2, *c*_1_=*c*_3_=−1, and *c*_2_=*c*_4_=1. Then the unconstrained dynamic model of this game is $\dot {P}=UEP+C$, where

$$UE=\left[ \begin{array}{cccc} 0 & 1 & 0 & 1 \\ -1 & 0 & -1 & 0 \\ 0 & 1 & 0 & 1 \\ -1 & 0 & -1 & 0 \\ \end{array} \right], C=\left(-1,1,-1,1\right)^{T}. $$

This game has a *P* ^∗^=(1/2,1/2,1/2,1/2)^*T*^∈(0,1)^4^, which satisfies *U* *E* *P* ^∗^+*C*=0. Matrix *UE* has a pair of pure imaginary eigenvalues which is *λ*_1_=2*i* and *λ*_1_=2*i*. The eigenvectors are *v*_1_=(0,1/2,0,1/2)^*T*^ and *v*_2_=(1/2,0,1/2,0)^*T*^ corresponding to *λ*_1_ and *λ*_2_. Let *P*(0)=*P* ^∗^+*k*_1_ *v*_1_+*k*_2_ *v*_2_. As long as *k*_1_ and *k*_2_ are sufficiently small, according to Theorem 1, the solution of the initial value problem of game 1 with *P*(0) can’t converge.

**Fig. 1:**
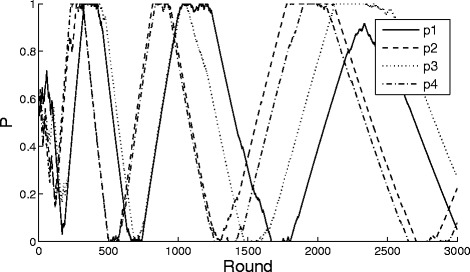
Agent dynamics of game satisfying the conditions of Theorem 1

In Fig. 1, the dynamic solution of the game with initial value *P*(0) is plotted, where *k*_1_=*k*_2_=0.1. Each of the four lines in Fig. 1 shows the strategy’s dynamic changing of each agent, respectively. We can see that the strategies of those agents do not converge. Obviously, the simulation results are consistent with the theoretical prediction.

### A convergence multi-agent Game

In this subsection, we consider a 4-player, two-action game. The game is defined as follows,

$${{R}_{i}}=\left[ \begin{array}{ll} 1 & 0 \\ 0 & 1 \\ \end{array} \right], i\in \{1,2,3,4\} $$

$$E=\left[ \begin{array}{cccc} 0 & 1/2 & 0 & 1/2\\ 1/2 & 0 & 1/2 & 0\\ 0 & 1/2 & 0 & 1/2 \\ 1/2 & 0 & 1/2 & 0\\ \end{array} \right] $$

Metrix *R*_*i*_,*i*∈{1,2,3,4} is the payoff matrix of agent *i*, and element *e*_*ij*_ of matrix *E* is the probability that player *i* selects player *j* in each interaction. In this game, we have *u*_*i*_=2 and *c*_*i*_=−1,*i*∈{1,2,3,4}. Then the unconstrained dynamic model of this game is $\dot {P}=UEP+C$, where

$$UE=\left[ \begin{array}{llll} 0 & 1 & 0 & 1 \\ 1 & 0 & 1 & 0 \\ 0 & 1 & 0 & 1 \\ 1 & 0 & 1 & 0 \\ \end{array} \right], C=\left(-1,-1,-1,-1\right)^{T}. $$

Because matrix *UE* is symmetrical, according to Theorem 4, the solution of the initial value problem of this game with any *P*(0)∈[0,1]^4^ will converge eventually.

**Fig. 2:**
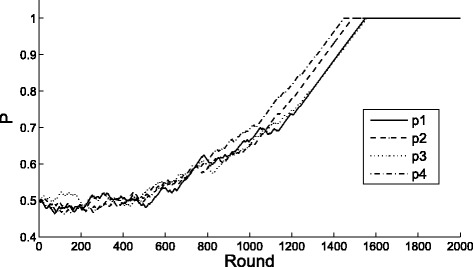
Agent dynamics of game satisfying the conditions of Theorem 4

Figure 2 illustrates dynamics of the PHC learners’ strategy for the game with initial value initial value *P*(0)=(1/2,1/2,1/2,1/2)^*T*^. Each of the four lines in Fig. 2 shows the strategy’s dynamic changing of each agent, respectively. We can see that the strategies of those agents converge eventually, which are consistent with the theoretical prediction.

## Conclusion

In this work, we proposed a multiagent social learning framework to model the behavior of agent in biologic environment, and theoretically analyzed the dynamics of multiagent social learning framework using non-linear dynamic theories. We present some sufficient conditions about convergence or non-convergence and prove them by the theoretically analysis. It can be used to predict the convergence of the system. Experimental results show that the predictions of our dynamic model are consistent with the simulation results.

As future work, more extensive study of the dynamics of multiagent social learning framework with *PHC* learners is needed. Other worthwhile directions include to improve the *PHC* algorithm, to develop more realistic multiagent social learning framework to model the realistic interactions among cells in biologic environments, and to achieve better convergence performance based on our theoretical findings.
